# Transcriptional Modulation of the Hippo Signaling Pathway by Drugs Used to Treat Bipolar Disorder and Schizophrenia

**DOI:** 10.3390/ijms22137164

**Published:** 2021-07-02

**Authors:** Bruna Panizzutti, Chiara C. Bortolasci, Briana Spolding, Srisaiyini Kidnapillai, Timothy Connor, Mark F. Richardson, Trang T. T. Truong, Zoe S. J. Liu, Gerwyn Morris, Laura Gray, Jee Hyun Kim, Olivia M. Dean, Michael Berk, Ken Walder

**Affiliations:** 1 Institute for Innovation in Physical and Mental Health and Clinical Translation, School of Medicine, Deakin University, IMPACT, Geelong 3220, Australia; b.panizzuttiparry@deakin.edu.au (B.P.); chiara.b@deakin.edu.au (C.C.B.); briana.spolding@deakin.edu.au (B.S.); srisaiyini.kidnapillai@med.lu.se (S.K.); timothy.connor@deakin.edu.au (T.C.); truongtra@deakin.edu.au (T.T.T.T.); zoe.liu@deakin.edu.au (Z.S.J.L.); activatedmicroglia@gmail.com (G.M.); l.gray@deakin.edu.au (L.G.); jee.kim@deakin.edu.au (J.H.K.); o.dean@deakin.edu.au (O.M.D.); michael.berk@deakin.edu.au (M.B.); 2 Genomics Centre, School of Life and Environmental Sciences, Deakin University, Burwood 3125, Australia; m.richardson@deakin.edu.au; 3 Florey Institute for Neuroscience and Mental Health, University of Melbourne, Parkville 3052, Australia; 4 Department of Psychiatry, Royal Melbourne Hospital, University of Melbourne, Parkville 3052, Australia; 5 Centre of Youth Mental Health, University of Melbourne, Parkville 3052, Australia; 6 Orygen Youth Health Research Centre, Parkville 3052, Australia

**Keywords:** Hippo pathway, psychotropic drugs, bipolar disorder, schizophrenia, inflammation, drug repurposing, connectivity map, psychiatry, neuroscience

## Abstract

Recent reports suggest a link between positive regulation of the Hippo pathway with bipolar disorder (BD), and the Hippo pathway is known to interact with multiple other signaling pathways previously associated with BD and other psychiatric disorders. In this study, neuronal-like NT2 cells were treated with amisulpride (10 µM), aripiprazole (0.1 µM), clozapine (10 µM), lamotrigine (50 µM), lithium (2.5 mM), quetiapine (50 µM), risperidone (0.1 µM), valproate (0.5 mM), or vehicle control for 24 h. Genome-wide mRNA expression was quantified and analyzed using gene set enrichment analysis (GSEA), with genes belonging to Hippo, Wnt, Notch, TGF- β, and Hedgehog retrieved from the KEGG database. Five of the eight drugs downregulated the genes of the Hippo pathway and modulated several genes involved in the interacting pathways. We speculate that the regulation of these genes, especially by aripiprazole, clozapine, and quetiapine, results in a reduction of MAPK and NFκB pro-inflammatory signaling through modulation of Hippo, Wnt, and TGF-β pathways. We also employed connectivity map analysis to identify compounds that act on these pathways in a similar manner to the known psychiatric drugs. Thirty-six compounds were identified. The presence of antidepressants and antipsychotics validates our approach and reveals possible new targets for drug repurposing.

## 1. Introduction

The Hippo pathway is a signaling cascade that integrates a broad range of different biological, chemical, and mechanical cues to control several cellular processes through its downstream effectors YAP (yes-associated protein) and transcriptional co-activator with PDZ-binding motif (TAZ) [1,2]. It was first discovered in Drosophila melanogaster, where a genetic mutation in one of its core components (Hippo/*Hpo*) was associated with tissue overgrowth of the eyes, wings, and limbs, a phenotype that gave name to the pathway [1]. The Hippo pathway regulates cell proliferation, differentiation, and spatial patterning governing organ size, tissue homeostasis, and regeneration, and is highly conserved from Drosophila to mammals [1,2].

The canonical Hippo signaling pathway is a key regulator of organ size and tissue remodeling [2]. The non-canonical Hippo signaling pathway, due to its diverse interplay with a variety of signaling cascades, i.e., TGF-β, Wnt, Hedgehog (HH), and Notch signaling pathways [3], has been associated with diversified mechanisms according to different microenvironments [4]: neurogenesis [5], neuronal development [6], neuronal dendritic field formation [7,8], and, more recently, neuroinflammation [9] and immunology [10]. In the central nervous system, the Hippo pathway has been associated with the response to neuroinflammation [11], dendrite development and organization [7], the balance between apoptosis and proliferation of the neural progenitor cell pool [12], glioma proliferation [13], Huntington’s disease [14], and Alzheimer’s disease [15].

Due to its role in both healthy and pathologic processes in the central nervous system and its crosstalk with other signaling pathways, it is unsurprising that the genes involved in the Hippo pathway have recently been associated with various psychiatric conditions [16,17,18,19]. Indeed, Liu and colleagues [18], identified positive regulation in the transcription of Hippo pathway genes in post-mortem prefrontal cortex of bipolar disorder (BD) patients as significantly enriched compared to healthy controls. Together with 30 hub genes, including *YAP*, the authors suggested that this pathway might have important implications in understanding the pathophysiology of BD, and could be a source of new targets for treatment. Further, the Hippo pathway genes were also reported as hypermethylated in a twin affected with BD compared with the non-affected twin [16]. Although the Hippo pathway was not differentially methylated in a second pair of twins, the authors proposed that the patient-specific differences might reflect the effects of antipsychotic medications, resulting in hyper/hypomethylation differences. In addition, different components of the Hippo pathway appear to be targeted by chlorpromazine, an antipsychotic used to treat schizophrenia (SZ), leading to apoptosis in cancer cells [20]. Valproic acid used to treat BD is reported to interact with the Hippo pathway through RASSF1A in myeloid leukemia, allowing YAP to associate with p73 and induce the expression of pro-apoptotic genes [21].

Therefore, this study aimed to evaluate the effects of commonly prescribed psychoactive drugs used in treating affective disorders (BD and SZ) on the expression of genes in the Hippo pathway. We expect these medications to downregulate the genes involved in the Hippo pathway, as upregulation of the Hippo pathway was previously observed in people with BD compared to healthy controls [18].

## 2. Results

### 2.1. GSEA

The list of genes involved in the Hippo pathway was extracted from the KEGG database, and the acute effects of the eight drugs on these genes was analyzed by GSEA. Five of the eight drugs, amisulpride, aripiprazole, clozapine, quetiapine, and risperidone, significantly downregulated genes in the Hippo pathway, as shown in Table 1.

### 2.2. Hippo Core Genes and Transcription Factors

**Figure 1:**
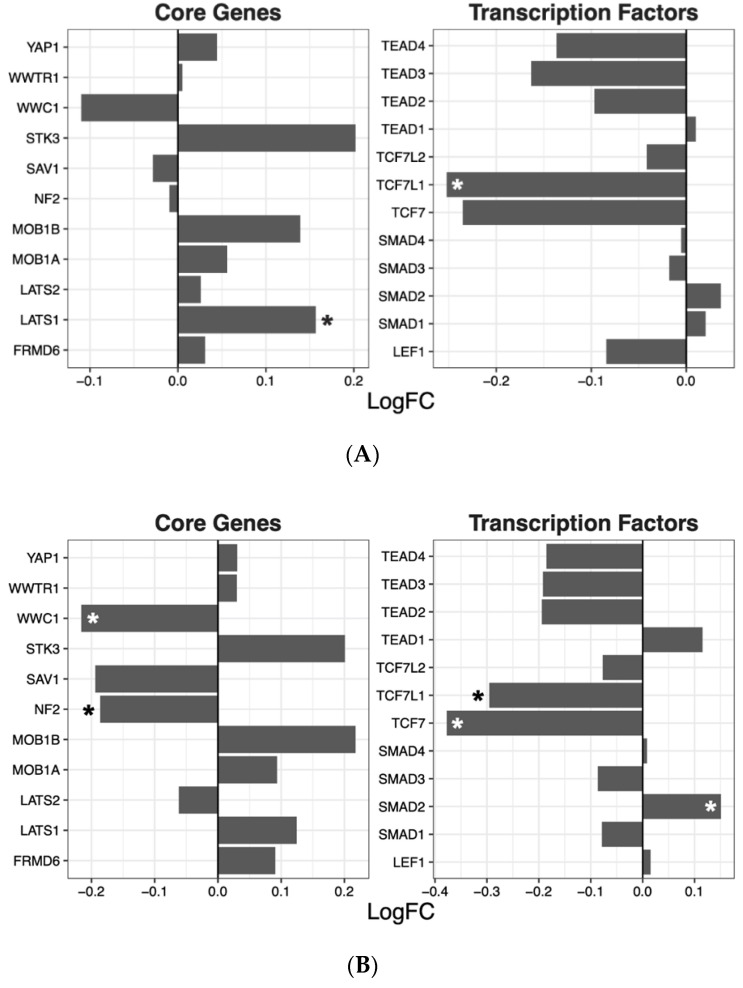

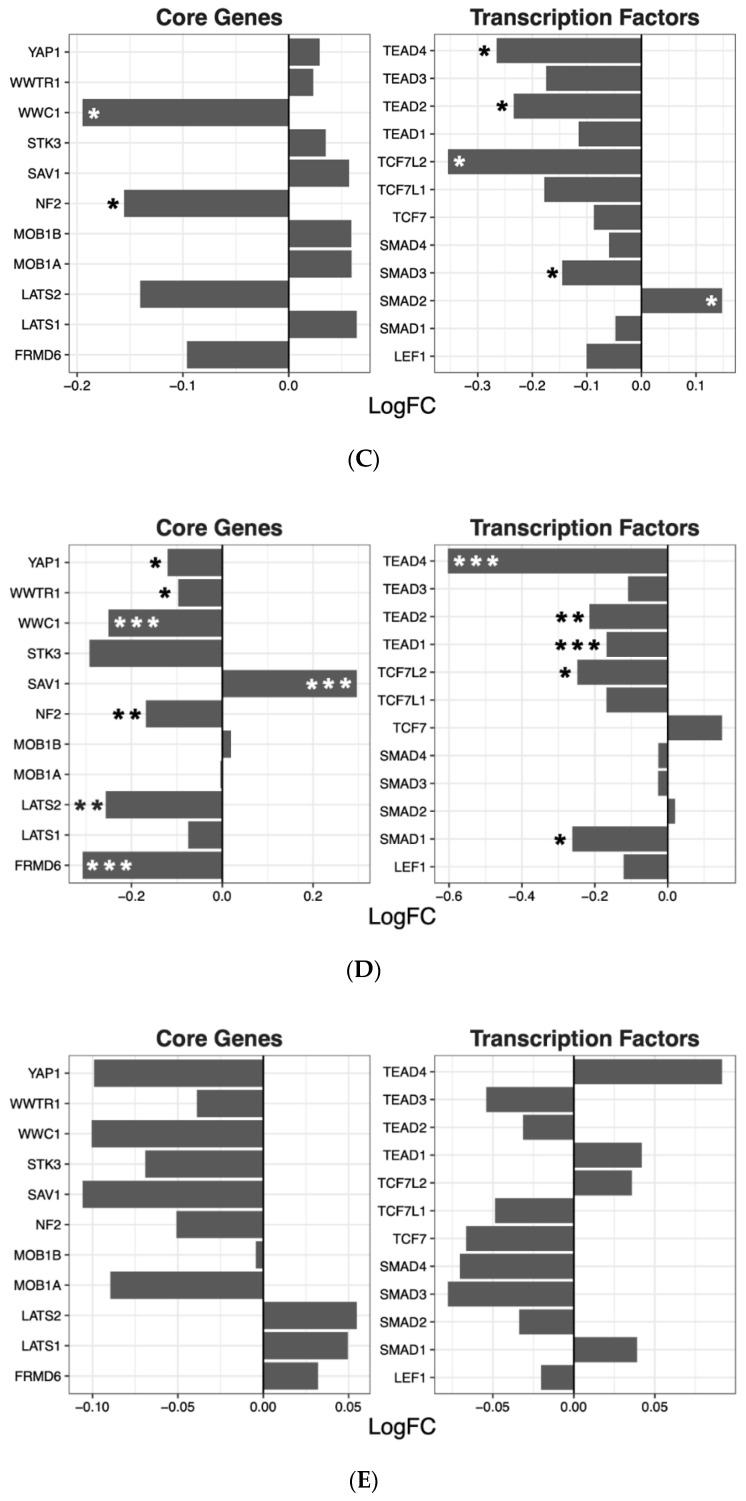
Effects of five drugs in the core genes and transcriptional factors of the Hippo pathway. (**A**) Amisulpride. (**B**) Aripiprazole. (**C**) Clozapine. (**D**) Quetiapine. (**E**) Risperidone * *p* < 0.05, ** *p* < 0.005, and *** *p* < 0.001.

Having ascertained that five of the psychoactive drugs significantly altered the expression of genes in the Hippo pathway, we further investigated the effects of these drugs on a smaller set of genes. This smaller set of genes included the core genes of the Hippo pathway and its transcription factors, which are the critical effectors of the pathway (Figure 1 A–E).

Amisulpride, aripiprazole, clozapine, and quetiapine significantly reduced the expression of the transcription factors involved in the Hippo pathway (*p* = 0.007, *p* = 0.025, *p* = 0.001, and *p* = 0.008, respectively). Risperidone and quetiapine downregulated the core genes of the Hippo pathway (*p*= 0.03 and *p* = 0.02, respectively), and amisulpride (*p* = 0.05) showed a tendency to downregulate the core genes of the Hippo pathway.

### 2.3. Interacting Pathways

The Hippo signaling pathway interacts extensively with other closely related signaling pathways, including the TGF-β, WNT, Notch, and Hedgehog signaling pathways. Therefore, the effects of these drugs were further investigated on these pathways (Table 2).

**Figure 2:**
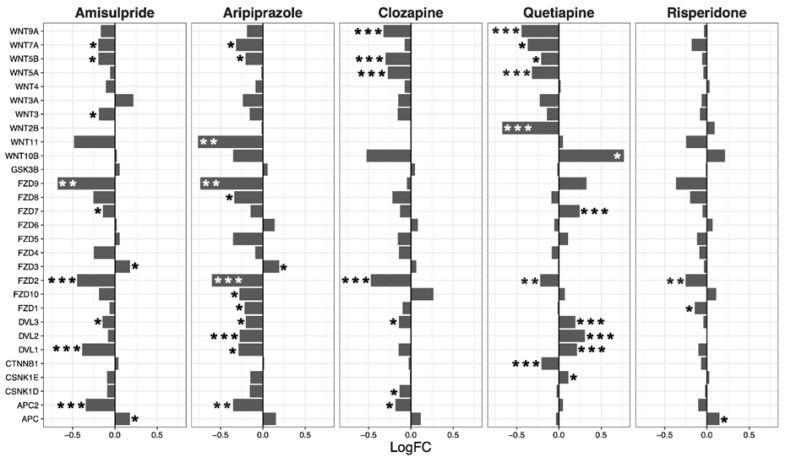
Wnt signaling pathway genes regulated by psychotropic drug treatment in NT2-N cells expressed as log fold change relative to vehicle-treated cells. * *p* < 0.05, ** *p* < 0.005, and *** *p* < 0.001.

The Wnt signaling pathway was transcriptionally downregulated by amisulpride (*p* = 0.0018), aripiprazole (*p* = 4.80 × 10^−6^), clozapine (*p* = 0.0013), and risperidone (*p* = 0.02; Table 2). These four drugs significantly regulated several individual genes in the Wnt pathway. While quetiapine also positively or negatively affected the transcription of a number of genes in the pathway, the overall effect of quetiapine on the Wnt signaling pathway was not significant (Figure 2).

**Figure 3:**
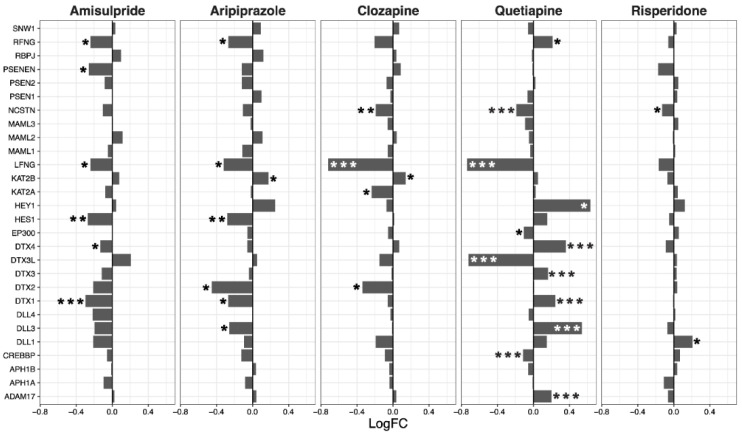
Notch signaling pathway genes regulated by psychotropic drug treatment in NT2-N cells expressed as log fold change relative to vehicle-treated cells. * *p* < 0.05, ** *p* < 0.005, and *** *p* < 0.001.

Overall, amisulpride (*p* = 0.0032), aripiprazole (*p* = 0.043), and clozapine (*p* = 0.0033) reduced the expression of genes in the notch signaling pathway. Again, quetiapine increased or decreased the expression of several genes in the pathway, but did not have a significant overall effect (Figure 3).

**Figure 4:**
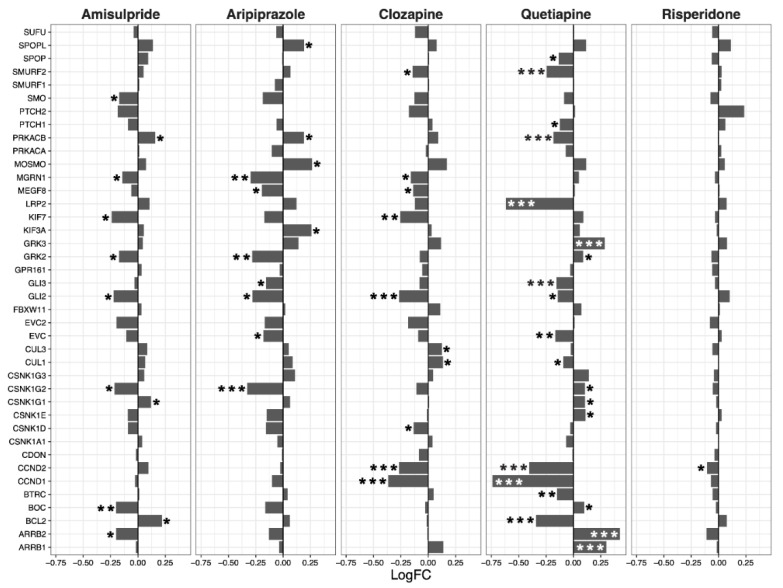
Hedgehog signaling pathway genes regulated by psychotropic drug treatment in NT2-N cells expressed as log fold change relative to vehicle-treated cells. * *p* < 0.05, ** *p* < 0.005, and *** *p* < 0.001.

Expression of genes in the hedgehog signaling pathway was decreased overall by clozapine (*p* = 0.025), and tended to be reduced by aripiprazole (*p* = 0.07, Figure 4). Clozapine significantly reduced the expression of *CCND1*, *CCND2*, and *GLI2* (Figure 4).

**Figure 5:**
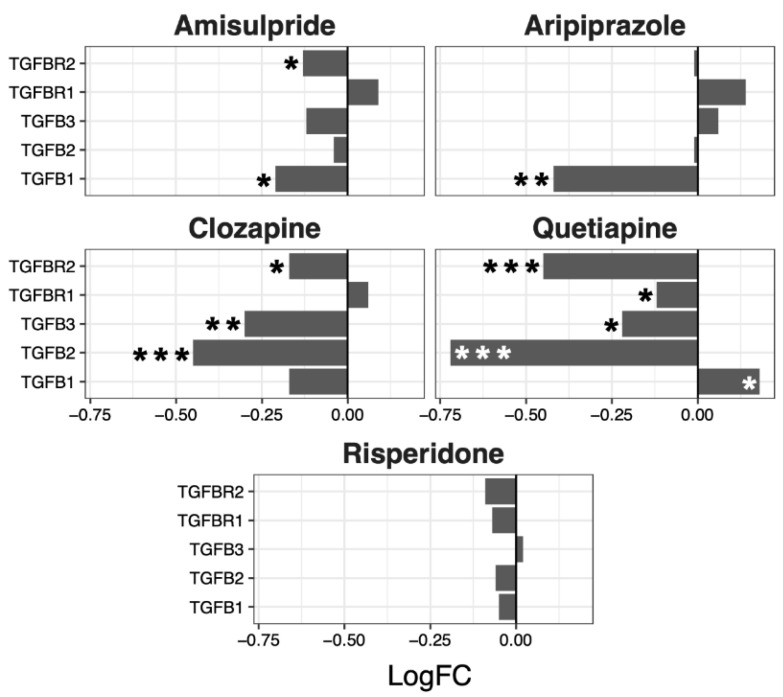
TGFβ signaling pathway genes regulated by psychotropic drug treatment in NT2-N cells expressed as log fold change relative to vehicle-treated cells. * *p* < 0.05, ** *p* < 0.005, and *** *p* < 0.001.

Although no overall significant effects were observed in the regulation of the TGF-β pathway, several genes were up- or downregulated by the different drugs, with a tendency to be downregulated by clozapine (*p* = 0.07) and risperidone (*p* = 0.06) (Figure 5).

### 2.4. CMap

The genes in these signaling pathways that were regulated by the five drugs in the same direction (n = 22 genes with evidence of mean log fold change compared with the vehicle of *p* < 0.1; Table S1) were submitted for CMap analysis, which identified 36 drugs with a positive CMap enrichment score of >0 and *p* < 0.05 (Table 3).

## 3. Discussion

Consistent with our hypothesis, five drugs commonly used to treat BD and schizophrenia (amisulpride, aripiprazole, clozapine, quetiapine, and risperidone) significantly downregulated the expression of Hippo signaling pathway genes, together with differential effects of each drug on various interacting pathways. These results complement reports of upregulation of these sets of genes in samples derived from patients with BD [18,19], possibly indicating a targeted effect of these drugs to revert Hippo-related immune activation and inflammation involved in the pathophysiology of affective disorders.

### 3.1. Aripiprazole and Clozapine 

The atypical antipsychotics aripiprazole and clozapine modulate dopaminergic receptors, as well as a range of other targets [22]. Our results show that these medications also modulate the Hippo signaling pathway genes, that is, they contribute to the downregulation of *NF2* and *WWC1* genes, accompanied by the downregulation of the Wnt and Notch pathways.

NF2 plays an indispensable role in the recruitment of MST1/2 and LATS1/2 to the plasma membrane, which enables LATS1/2 phosphorylation and repression of YAP/TAZ and MOB1 [23,24,25]. Complete abrogation of NF2 activity results in an inability to suppress the activity of YAP/TAZ [26,27]. WWC1, WWC2, and WWC3 are another family that positively regulates the Hippo pathway via the activation of LATS1/2 [28,29]. Importantly, loss of WWC activity results in the nuclear translocation of YAP/TAZ, enabling their activation as transcription factors [28,29].

The downregulation of *NF2* and *WWC1* observed following treatment with aripiprazole and clozapine could lead to YAP/TAZ nuclear translocation and its non-canonical activities: inhibition of TGF-β signaling [30,31] and modulation of the activity of the Wnt and Notch pathways [32,33]. YAP/TAZ activation is also associated with reduced activity of NFκB and pro-inflammatory cytokine production. This appears to be mediated by the inhibition of proteins and enzymes involved in downstream signaling cascades following pattern-recognition receptors (PPRs) activation [34,35] (reviewed [36]).

In addition, clozapine also downregulated the Hh pathway, accompanied by significant downregulation of *GLI2*, a downstream effector of SHH. The Hh signaling pathway has been associated with maintaining the stem propriety in cells in the hippocampus and cerebellum [37,38]. Similar results showed the inhibition of the Hh pathway and downregulation of *GLI2* following treatment with clozapine, haloperidol, and chlorpromazine in ShhL2, C3H10T1/2, and T98G cells [39]. The finding that drugs used to treat schizophrenia can modulate the Hh pathway raises the possibility that alterations in Hh signaling might contribute to the disease aetiology, as suggested by Boyd et al. in 2015 [40].

### 3.2. Amisulpride

Similar to aripiprazole and clozapine, amisulpride also significantly downregulated the Wnt and Notch signaling pathways. The overall downregulation exerted by amisulpride in the Hippo signaling pathway seems to be primarily driven by effects on the expression of the transcription factors, particularly TCFs and TEADS (Figure 1A). T-cell factor/lymphoid enhancer-binding factor (TCF/LEF) proteins (TCFs) are the main downstream effectors of Wnt signaling [41] and associate with β-catenin in the nucleus to activate transcription of Wnt signaling target genes. The upregulation of *LATS1* following treatment with amisulpride could lead to YAP/TAZ sequestration to the cytoplasm, which binds to β-catenin differently to nuclear YAP/TAZ, preventing translocation to the nucleus and therefore inhibiting Wnt signaling [42]. TEA domain transcription factors (TEADs) exert multiple regulatory effects on signaling pathways other than the Hippo, and the activity of these signaling pathways influences the activity of TEADs. For example, upregulation of the Wnt pathway results in the activation of TEADs whose increased transcriptional activity inhibits Wnt signaling, thus engaging a negative feedback system [43,44]. TGFβ mediated signaling also increases levels and activity of TEADs, but in this instance, increased TEAD activity increases TGFβ signaling, forming a positive feedback loop [45,46]. Finally, increased activity of TEADs also acts as a negative regulator of Hippo signaling by increasing the levels and activity of LATS and NF2 [47,48] (reviewed [49]).

### 3.3. Quetiapine and Risperidone

Quetiapine and risperidone have different mechanisms of action compared to the medications above [22], which may correspond with their effects on the Hippo signaling pathway. Quetiapine presented a broad range of effects with significant downregulation of the core Hippo genes and the transcriptional factors of the Hippo pathway, complemented by a variety of significant but multidirectional (up- and downregulated) effects on the interacting pathways. Although the precise effects of quetiapine remain to be elucidated, we speculate that it could involve effects besides the NF2 effects described above, as NF2 activity also upregulates numerous pro-inflammatory signaling pathways, including PI3K-AKT, MAPK, and ERK [27,50].

The opposite was found for risperidone, with the GSEA analysis indicating a trend for downregulation of genes in the Hippo signaling pathway, despite none of the genes reaching statistical significance.

### 3.4. Summary

Downregulation of *NF2* and *WWC1* was observed following administration of aripiprazole, clozapine, and quetiapine. Likely consequences include reduced inflammatory signaling mediated by MST1/2 and NF2, and reduced MAPK and NFκB activity levels following upregulation of YAP/TAZ. Aripiprazole also downregulates T-cell factors, potentially compromising Wnt signaling [51].

Several authors have demonstrated that MST1/2 directly regulates many aspects of immune activation and the activity of inflammatory pathways [52,53]. Examples of pathways upregulated include MAPK (mitogen-activated protein kinase) and p53 [36]. MST1/2 also plays a major role in Toll-like receptor (TLR) signaling and subsequent activation of NFκB and pro-inflammatory cytokine production [54,55]. Similarly, *YAP* deletion in astrocytes induced JAK-STAT pathways, inducing reactive astrogliosis, microglial activation, and BBB dysfunction in mice [56].

**Figure 6:**
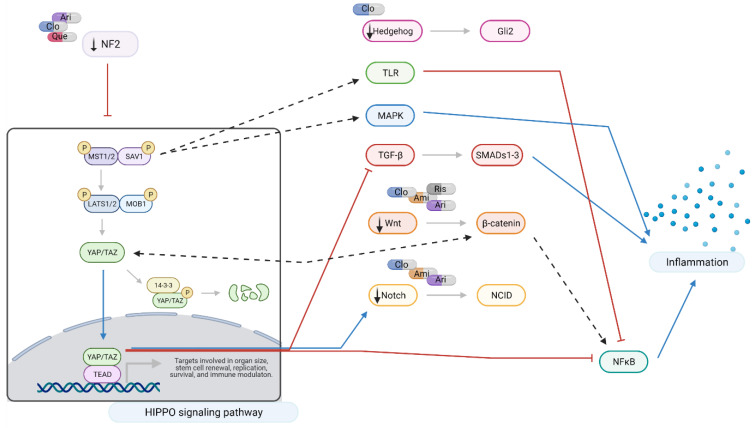
Hippo signaling at a glance: effects of amisulpride, aripiprazole, clozapine, quetiapine, and risperidone. Legend: Ami = amisulpride; Ari = aripiprazole; Clo = clozapine; Que = quetiapine; Ris = risperidone; red arrows = inhibition; blue arrows = activation; dotted arrows = modulation; and black arrows = drugs action. In neuronal-like NT2 cells, inhibition of Hippo pathway reduces the production of pro-inflammatory cytokines through the crosstalk with TRL, MAPK, TGF-β, Wnt, and Notch signaling pathways. Aripiprazole, clozapine, and quetiapine downregulate NF2, leading to activation and nuclear translocation of YAP/TAZ which results in a reduction in NFκB and TGF-β signaling. NF2 also closely interacts with Hedgehog, TGF-β, Wnt, and Notch pathways. Modulation of MST1/2 expression also results in reduced pro-inflammatory signaling through Toll-like receptor (TRL) and MAPK signaling. Amisulpride, aripiprazole, clozapine, and risperidone downregulate Wnt signaling interfering with NFκB and pro-inflammatory cytokine production. Created with BioRender.com.

When considered as a whole, the net effects of these changes would reduce pro-inflammatory signaling mediated by MAPK and NFκB. This is of interest as aripiprazole [57], quetiapine [58], and clozapine [59,60] are known to inhibit NFκB. The data produced in this study suggests that such inhibition may be achieved, at least in part, by effects on Hippo, Wnt, and TGFβ signaling (Figure 6).

### 3.5. Drug Repurposing

We used CMap to search for drugs that target the Hippo pathway genes similarly to the drugs we used to treat the NT2 cells. The CMap analysis identified 39 compounds with a *p*-value < 0.05 acting on the signaling pathway genes similarly to amisulpride, aripiprazole, clozapine, quetiapine, and risperidone (Table 3). Nine of these compounds have mechanisms associated with a reduction in NFκB signaling: ursolic acid [61], carbimazole [62], NS−398 [63], sodium phenylbutyrate [64], furazolidone [65], iloprost [66], ergocalciferol [67], clozapine [59], and triflupromazine [68].

For example, ursolic acid (UA) is a natural pentacyclic triterpenoid carboxylic acid present in various plants and is a recurrent component of our diet [69]. As part of traditional medicine, it is known for its antioxidant, anti-inflammatory, and anticancer properties [70]. Checker and colleagues [71] investigated the immunomodulatory effects of UA on T-cell activation following several stimuli, and showed that UA inhibits the activation of NFκB and other transcription factors, as well as MAP Kinases. The neuroprotective effects of UA have also been studied in depression, with effects being associated with dopamine 1 and 2 receptor activation [72]. Likewise, antioxidant effects by inducing antioxidant defenses were demonstrated in Alzheimer’s disease [73]. The possible beneficial effects of UA in neurodegenerative and psychiatric conditions were recently reviewed [74,75].

The identification of the antipsychotics, including clozapine, piperacetazine, and triflupromazine by this CMap analysis adds weight to our contention that this is a valuable methodology to identify drugs which act in a similar manner to the drugs we tested, and gives confidence that they represent potential targets for repurposing to treat psychotic and affective disorders.

## 4. Materials and Methods

### 4.1. Cell Culture

NT2 human teratocarcinoma cells (CVCL_0034, ATCC, Manassas, VI, USA) were cultured, maintained and differentiated as previously described [76]. Briefly, to generate an enriched culture of differentiated neuronal cells, NT2 cells were treated with retinoic acid (Sigma-Aldrich, Sydney, Australia) at 1 × 10^−5^ M for 28 days. Following retinoic acid treatment, cells were plated for experiments in 24-well plates at 2 × 10^5^ cells/well and treated with mitotic inhibitors (1 µM cytosine and 10 µM uridine, Sigma-Aldrich) for 7 days.

Once enriched cultures of differentiated neuronal cells (NT2-N) were obtained, the cells were treated with drugs commonly prescribed in psychiatry (amisulpride (10 µM), aripiprazole (0.1 µM), clozapine (10 µM), lamotrigine (50 µM), lithium (2.5 mM), quetiapine (50 µM), risperidone (0.1 µM), or valproate (0.5 mM); Sigma-Aldrich) or the appropriate vehicle control (DMSO 0.2% or Milli-Q water 0.5%) for 24 h (n = 4–5 per group). The dosages chosen for the in vitro treatment were determined based on previous dose–response studies so that, when used in combination, no single drug dominated the overall effect on gene expression nor affected cell viability. Such doses were carried out throughout the following projects [77]. The experimental procedure used was carried out throughout several projects in our lab, with previous publications also showing differences in gene expression after 24 h treatment [76,78]. In addition, the use of psychotropic drugs in vitro has shown alterations on gene expression after 24 h, and as early as after 1 h treatment [79,80].

### 4.2. Gene Expression

Following the 24-h drug treatment, cells were harvested using Trizol, and total RNA was extracted using RNeasy^®^ mini kits (Qiagen, Melbourne, Australia) and quantified by spectrophotometry (NanoDrop 1000 Thermo Fisher Scientific, Waltham, MA, USA). The quality of the extracted RNA was evaluated using an Agilent 2100 Bioanalyzer (Agilent Technologies, Melbourne, Australia). RNAseq libraries were prepared from 1 ug of total RNA using a TruSeq RNA samples Preparation kit (Illumina, Victoria, Australia). The libraries were sequenced using a HiSeq 2500 flow cell (50 bp single end reads; Illumina) according to the manufacturer’s instructions.

### 4.3. Genome-Wide Gene Expression Analysis

The raw data were obtained in fastq format, and processed using the Deakin Genomics Centre RNAseq alignment and expression quantification pipeline (https://github.com/m-richardson/RNASeq_pipe, accessed on 1 July 2017). In summary, this involves: Raw read quality filtering and adapter trimming (ILLUMINACLIP:2:30:10:4, SLIDINGWINDOW:5:20, AVGQUAL:20 MINLEN:36) with Trimmomatic v35 [81], and alignment to the reference genome using STAR v2.5 in 2-pass mode (Human genome version GRCh38) [82]. The expression was quantified at the gene level, and individual sample counts were collated into a m × n matrix for differential abundance testing. Normalization (TMM) and removal of low expressed gene were performed using edgeR [83] in R [84] following the edgeR manual (<1 cpm in n samples, where n is the number of samples in the smallest group for comparison). Differential gene expression analysis was assessed using edgeR in R, and the Benjamini–Hochberg [85] corrected p-values were calculated to account for multiple testing. Genes with *p*-values of <0.05 were considered to be differentially expressed.

### 4.4. Gene Set Enrichment Analysis (GSEA)

GSEA was deployed using the R package cluster Profiler [86] from Bioconductor [87], with gene lists pre-ranked based on the sign of log fold changes multiplied by the log10-transformed of *p*-values from the differential analysis. The Kyoto Encyclopedia of Genes and Genomes (KEGG) database was used as a reference database, and only gene sets with sizes ranging from 3 to 800 genes inclusive were considered. The resulting tables had enrichment scores and *p*-values calculated from 10,000 permutations, with accompanying false discovery rate *q*-values adjusted for multiple testing.

### 4.5. Interacting Pathways

The list of genes belonging to the Wnt signaling pathway, Notch signaling pathway, Hedgehog signaling pathway, Transforming Growth Factor Beta (TGF-β) signaling pathway were obtained from KEGG, and then tested for overall transcriptional regulation by the drugs. The distribution of logFC data was checked for normality using Kolmogorov–Smirnov tests. Drug treatment groups were compared against their respective controls using independent samples t-tests for normally distributed data and Mann–Whitney U tests for data not normally distributed.

### 4.6. Connectivity Map (CMap)

Genes in the Hippo pathway that were significantly increased or decreased by 4 or more of the drugs were assigned a tag ID recognizable by CMap using Human Genome U133 Plus 2.0 Array on Affymetrix (https://www.affymetrix.com/site/mainPage.affx; Thermo Fisher Scientific, accessed on 1 November 2020). The IDs were collated in sets of “up” or “down” regulated genes and submitted to CMap. The similarities between the gene expression patterns induced by the compounds in the CMap database and those caused by the psychoactive drugs used in this project were reported as connectivity scores ranging from +1 (increased similarity) to −1 (inverse similarity).

## 5. Conclusions

To our knowledge, this is the first study to evaluate the effects of mood stabilizers and antipsychotics on the expression of genes involved in the Hippo pathway and its interacting pathways. Remarkably, the effects and routes described here were previously observed in oncology, due to the role of the Hippo signaling pathway in tumorigenesis. In this context, further research investigating the effects of these genes and pathways in psychiatry is necessary. We acknowledge some limitations of our study. All experiments were conducted in neuronal-like cells without the induction of any disease state model; therefore, underlying disease-specific pathophysiological processes might alter the drug effects. In addition, we tested a single dose of each drug and measured its acute effects, hence these findings do not capture chronic administration effects or drug-drug interaction during polypharmacy.

The identification of new pathways associated with psychiatric conditions that can be targeted by known pharmacological treatments can highlight an opportunity for the development of new treatment options for these debilitating conditions. This represents an opportunity to offset the lack of mechanistic understanding in BD and SZ, as well as other psychiatric conditions, and the curtailment in research and development by major pharmaceutical companies [88]. The use of differential gene expression and connectivity maps in this study identified a number of drugs with known effects on proposed pathological mechanisms associated with psychiatric disorders. Some of the other drugs identified could be further explored as potential therapeutic options for psychiatric disorders.

## Figures and Tables

**Table 1:** Effects of psychoactive drugs on KEGG Hippo pathway gene expression.

Drug	ES	NES	*p*-Value	*q*-Value
Clozapine	−0.61	−2.09	**0.00013**	**0.0012**
Aripiprazole	−0.54	−2.04	**0.00015**	**0.0044**
Risperidone	−0.49	−1.92	**0.00024**	**0.0049**
Quetiapine	−0.60	−1.79	**0.00027**	**0.0052**
Amisulpride	−0.45	−1.61	**0.00085**	**0.016**
Lithium	−0.40	−1.32	**0.048**	0.21
Lamotrigine	−0.29	−1.15	0.14	0.35
Valproate	0.31	0.89	0.67	0.66

Medications listed based on the *p*-value, lowest to highest. Abbreviations: ES = enrichment score; NES = normalized enrichment score.

**Table 2:** The effects of the drugs on genes involved in interacting pathways.

	Wnt			Notch			Hedgehog			TGF-β		
	LogFC		*p*-Value	LogFC		*p*-Value	LogFC		*p*-Value	LogFC		*p*-Value
	Mean	SEM		Mean	SEM		Mean	SEM		Mean	SEM	
Amisulpride	−0.13	0.04	**0.0018**	−0.08	0.03	**0.0032**	−0.03	0.02	0.18	−0.08	0.05	0.19
Aripiprazole	−0.21	0.04	**4.80 × 10^−6^**	−0.07	0.03	**0.043**	−0.04	0.02	0.07	−0.05	0.1	0.66
Clozapine	−0.11	0.03	**0.0013**	−0.08	0.03	**0.0033**	−0.05	0.02	**0.025**	−0.21	0.08	0.07
Quetiapine	−0.02	0.05	0.64	0.02	0.05	0.63	−0.04	0.03	0.26	−0.27	0.15	0.15
Risperidone	−0.06	0.02	**0.02**	−0.01	0.02	0.72	0	0.01	0.67	−0.05	0.02	0.06

Abbreviations: LogFC= logarithmic fold change; SEM = standard error of the mean.

**Table 3:** Compounds that affect the expression of the gene expression signature genes similar to the five drugs that affected the Hippo pathway.

Rank	CMap Name	Enrichment	*p*-Value	Class
1	Ursolic acid	0.91	0.00008	Triterpenoid
2	Levothyroxine sodium	0.89	0.00014	Thyroid hormones
3	Ajmaline	0.94	0.00022	Antiarrhythmic agent
4	5707885	0.87	0.00044	Unknown
6	Carbimazole	0.90	0.00182	Imidazole–thyroid function
7	0297417–0002b	0.88	0.00324	Unknown
9	Ns-398	0.87	0.00463	COX-2 inhibitor (anti-inflammatory)
10	Cefapirin	0.78	0.00473	Antibiotic
11	Estrone	0.77	0.00539	Estrogen steroid
12	Strophanthidin	0.77	0.00561	Cardiac glycoside
17	Picotamide	0.68	0.00937	Platelet aggregation inhibitor
20	Maprotiline	0.72	0.0117	Tetracyclic antidepressant
22	Monastrol	0.53	0.01256	Antimitotic agent
24	Sr-95639a (Aminopyridazine)	0.72	0.01331	Muscarinic agonist
28	Benzethonium chloride	0.78	0.02097	Antiseptics and Disinfectants
30	Ciprofibrate	0.68	0.0228	Fibrate (lipid-lowering agent)
31	5255229	0.90	0.02286	Unknown
32	Methazolamide	0.68	0.02316	Carbonic anhydrase inhibitors
33	Bumetanide	0.68	0.02379	Diuretic
37	Sodium phenylbutyrate	0.52	0.02663	Urea cycle disorder treatment agents
38	Tenoxicam	0.67	0.02795	NSAID (Nonsteroidal anti-inflammatory drug)
40	Ah-23848	0.76	0.0291	Prostanoid EP4 antagonist
41	Furazolidone	0.66	0.03058	Oxazolidine–antibiotic agent
42	Iloprost	0.75	0.03201	Vasodilator
43	Minoxidil	0.60	0.03344	Vasodilator
46	Hyoscyamine	0.59	0.03475	Anticholinergic/antispasmodic
45	Prestwick-642 (Epicatechin)	0.65	0.03475	Catechin–antioxidant flavonoid
48	Dihydrostreptomycin	0.59	0.03529	Antibiotic
49	Nisoxetine	0.65	0.03539	Antidepressant -SNRI
50	Ergocalciferol	0.65	0.03589	Vitamin D analogue
52	Ceforanide	0.65	0.03804	Antibiotic
55	Pheniramine	0.58	0.04175	Antihistamine
56	Ifenprodil	0.64	0.04245	NMDA receptor antagonist
57	Pha-00745360	0.46	0.04312	Unknown
58	Clozapine	0.32	0.04422	Atypical antipsychotic
60	Diphemanil methylsulfate	0.57	0.04552	Muscarinic antagonist
63	Piperacetazine	0.63	0.04729	Antipsychotic prodrug
66	Trifluoperazine	0.63	0.04882	Phenothiazine antipsychotic
68	Lidoflazine	0.71	0.04974	Vasodilator

## Data Availability

Data available from author upon reasonable request.

## Supplementary Materials

The following are available online at https://www.mdpi.com/article/10.3390/ijms22137164/s1.
