# Supplementary material for: Transcriptional Modulation of the Hippo Signaling Pathway by Drugs Used to Treat Bipolar Disorder and Schizophrenia
Source: Int J Mol Sci. 2021 Jul 2;22(13):7164. doi: 10.3390/ijms22137164 (PMC8268913; doi:10.3390/ijms22137164)
Supplement: Supplementary file 1 [file ijms-22-07164-s001.zip › ijms-1287510-supplementary.pdf]

International Journal of
Molecular Sciences

Supplementary Materials

Table S1. Genes in the HIPPO, Wnt, BMP, Notch and Hedgehog signaling pathways with evidence of overall downregulation by amisulpride, aripiprazole, clozapine, quetiapine, and risperidone ($p<0.05$). These genes were used in the CMap analysis.

		**LogFC**		
**Gene**	**Pathway**	**Mean**	**SEM**	**p-value**
APH1A	Notch	-0.07	0.02	**0.022**
BMP4	BMP	-0.15	0.07	**0.043**
CSNK1D	Wnt	-0.09	0.03	**0.035**
EVC2	Hedgehog	-0.12	0.04	**0.034**
FZD1	Wnt	-0.11	0.03	**0.035**
FZD2	Wnt	-0.40	0.07	**0.0050**
FZD4	Wnt	-0.13	0.03	**0.013**
FZD8	Wnt	-0.22	0.04	**0.0060**
GLI3	Hedgehog	-0.09	0.03	**0.031**
LFNG	Notch	-0.44	0.12	**0.023**
NCSTN	Notch	-0.14	0.02	**0.0020**
NF2	HIPPO	-0.11	0.04	**0.032**
SMAD3	HIPPO	-0.07	0.02	**0.034**
SMO	Hedgehog	-0.13	0.02	**0.0050**
TCF7L1	HIPPO	-0.19	0.04	**0.011**
TEAD2	HIPPO	-0.15	0.04	**0.016**
TEAD3	HIPPO	-0.14	0.03	**0.0060**
WNT3	Wnt	-0.15	0.02	**0.0010**
WNT5B	Wnt	-0.19	0.04	**0.0070**
WNT7A	Wnt	-0.23	0.05	**0.013**
WNT9A	Wnt	-0.23	0.07	**0.030**
WWC1	HIPPO	-0.17	0.03	**0.0040**
